# Association of socioeconomic status on return to work following primary total hip arthroplasty: a Danish population-based cohort study on 9,431 patients from 2008–2018

**DOI:** 10.2340/17453674.2025.43189

**Published:** 2025-03-10

**Authors:** Peter ALSING, Julie B PAJANIAYE, Martin G STISEN, Søren OVERGAARD, Erzsébet HORVÁTH-PUHÓ, Inger MECHLENBURG, Alma B PEDERSEN

**Affiliations:** 1 Department of Clinical Epidemiology, Aarhus University Hospital, Aarhus; 2 Department of Orthopaedic Surgery, Aarhus University Hospital, Aarhus; 3 Department of Dentistry and Oral Health, Aarhus University, Aarhus; 4 Department of Clinical Medicine, Aarhus University, Aarhus; 5 Department of Orthopaedic Surgery and Traumatology, Copenhagen University Hospital, Bispebjerg; 6 Department of Clinical Medicine, Faculty of Health and Medical Sciences, University of Copenhagen; 7 Center for Population Medicine, Aarhus University and Aarhus University Hospital; 8 Department of Public Health, Aarhus University, Aarhus; 9 VIA University College, Research Center for Rehabilitation, Aarhus, Denmark

## Abstract

**Background and purpose:**

Return to work (RTW) following primary total hip arthroplasty (THA) is important for patients and society. We aimed to investigate the association between markers of socioeconomic status (SES) and RTW after primary THA, and whether the association is influenced by sex, age, and comorbidity.

**Methods:**

Using Danish population-based registries we included 9,431 patients aged 18 to 59 years, undergoing primary THA for osteoarthritis from 2008–2018. Exposure was individual-level data on SES markers (education, income, and cohabitation). Work status information before and after THA was obtained from the Danish Register for Evaluation of Marginalization. We computed cumulative incidence of RTW up to 24 months after THA. The association between SES and RTW was analyzed using Cox regression by hazard ratios with 95% confidence intervals (CI).

**Results:**

The median time to RTW was 54 days. Cumulative incidence of RTW was 86% by 6 months and 93% by 24 months. The adjusted hazard ratio for RTW was 1.9 (CI 1.8–2.0) for high vs low education, 2.2 (CI 2.1–2.3) for high vs low income, and 1.3 (CI 1.3–1.4) for cohabiting vs living alone. Associations were stronger in male than female patients for all SES markers.

**Conclusion:**

Most patients returned to work within 24 months, with the largest proportion within 6 months. Markers of low SES were associated with delayed RTW, highlighting the importance of enhanced focus on THA patients in socially vulnerable positions to reduce health and financial implications of delayed RTW.

Osteoarthritis (OA) is a leading cause of disability and an economic burden worldwide [1]. Hip OA is the second most common type, resulting in decreased mobility, pain, and increased mortality [2,3]. For severe hip OA, total hip arthroplasty (THA) is an effective surgical intervention that relieves pain, improves function, and increases health-related quality of life [4]. Globally, the incidence of THA due to OA is projected to increase in the coming years [5,6].

About 20% of patients undergoing primary THA are < 60 years of age [5], indicating a large proportion of patients still in the workforce, and who need to return to work (RTW). RTW holds significant financial implications for patients and society. It contributes to improved general and mental health, and is as important a health determinant as exercise and diet [7]. Conversely, unemployment is associated with increased medication intake, mental disorders, and mortality [7]. Recent systematic reviews have reported wide variations globally in RTW rates following THA, as well as high degrees of heterogeneity in study design and quality [8,9]. The reasons for variation in RTW rates are poorly understood.

Socioeconomic status (SES) is a significant factor influencing health-related behaviors and outcomes [10], including those related to THA [11]. The impact of SES on RTW after THA is an area with limited and inconsistent findings [8]. Contemporary research in this area is important from a societal and clinical perspective regarding optimal resource allocation and to increase focus on socially vulnerable patients. As SES is closely related to age, sex, and comorbidity, it is furthermore important to examine these factors in relation to SES and RTW.

Therefore, we aimed to investigate the association between markers of SES and RTW following primary THA, and whether sex, age, and comorbidity influence this association.

## Methods

### Study design and setting

We conducted this nationwide population-based cohort study using Danish registries. All Danish residents have free and equal access to healthcare.

This study is reported according to STROBE guidelines.

### Data sources

All Danish residents receive a unique civil personalized registration number (CPR number) at birth or upon immigration, enabling individual-level linkage of data across multiple registries. Data linkage between the following registries was done: the Danish Hip Arthroplasty Registry (DHR), the Danish National Patient Registry (DNPR), the Danish Register for Evaluation of Marginalization (DREAM), Statistics Denmark, and the Danish Civil Registration System (CRS).

### Study population

The DHR was used to identify the study population of THA patients. It is regarded as a database with a high degree of validity [12], with data completeness ranging between 94% and 98% from 2008–2018, when compared with information from the DNPR [13]. Inclusion criteria were patients undergoing primary THA due to hip OA, from 2008–2018. Exclusion criteria were patients < 18 or ≥ 60 years of age, with the upper limit set to exclude patients planning for early retirement before the public retirement age of 65 years. Further exclusion criteria were having a status as retired in the DREAM 4 weeks prior to surgery, or missing data on any of the SES markers.

### Socioeconomic status

Statistics Denmark is a central authority on Danish statistics [14] and was used to obtain information on SES as exposure. SES was defined by 3 individual-level markers; education, income, and cohabiting status. Education was classified as low, defined as primary school or less, medium, defined as vocational education or medium-cycle higher education, and high, defined as a bachelor’s degree or higher, based on highest completed education at the time of THA. Income was calculated based on equivalized household income, with weighted estimates accounting for family composition, and variation of salary changes by calculating the mean annual household income during the 5 years prior to THA. Income, as an expression for material resources, was classified as low, medium, or high, corresponding to first, second, and third tertile, respectively. Cohabiting status was classified as cohabiting or living alone, with cohabiting being equivalent to high SES and defined as married couples, couples in general, or households consisting of more than 1 family.

### Return to work

The outcome was RTW. Employment status was collected from the DREAM, which consists of weekly information on social public transfer payments for all Danish citizens since 1991 and is considered a valid and useful source for research in public health [15]. Employer-paid sick leave, lasting 14 days post-THA, is not registered in DREAM.

The RTW was defined as having a status of working for 4 consecutive weeks after surgery in the DREAM database, a valid method to assess RTW using DREAM [16]. Working was defined as not receiving public transfer payments or receiving benefits related to education, modified jobs, or vocational rehabilitation. Time to RTW was assessed as the number of days from THA until whichever of the following occurred first: RTW, a competing event (retirement or death), censoring (emigration), or not RTW within the 24-month follow-up period. Each DREAM code from 2008–2018 was assessed and categorized regarding descriptive purposes, eligibility criteria, and outcome categories (Table S1, see Supplementary data).

### Covariates

Based on our analytical design shown in a directed acyclic graph (DAG) (Figure S1, see Supplementary data) and previous studies, we included several important covariates for investigating RTW following THA [17,18]. Sex and age were collected from the CRS. Sex was defined as male or female, and age was divided into categories of 18–44, 45–49, 50–54, and 55–59 years of age. Comorbidity history for all patients was retrieved from the DNPR, which holds complete nationwide coverage of data from Danish hospitals, including information on discharge diagnoses based on the International Classification of Diseases [19]. Information on comorbidity was retrieved up to 10 years prior to THA and assessed using the Charlson Comorbidity Index (CCI), which includes 19 diagnosis groups weighted by severity of each diagnosis in terms of mortality [20]. Based on the total CCI score, each patient was categorized into low, a CCI score of 0, medium, a score of 1–2, and high, a score of ≥ 3. Body mass index (BMI) was collected from the DHR and patients were categorized into non-obese (BMI < 30) and obese (BMI ≥ 30), as described in previous literature [17].

### Statistics

Descriptive statistics were presented with mean and standard deviation (SD) for continuous data if normally distributed; if not, as median and interquartile range (IQR). Confidence interval was presented as CI, defined as a 95% confidence interval. Categorical data was presented with proportion (n) and percentage. Time to RTW was presented as the median time (in days) and corresponding IQR, for the overall study population and for each marker of SES.

The cumulative incidence was calculated using the Aalen–Johansen method, with death and retirement considered as competing events. The information on death was obtained from the CRS [21]. Cumulative incidences were calculated at 1, 3, 6, 12, and 24 months after THA, both overall and for each marker of SES, and presented as percentages with corresponding CI along with cumulative incidence curves.

Associations between SES markers and RTW were analyzed using Cox proportional hazards regression models. Crude and adjusted hazard ratios (aHR) were calculated with corresponding 95% CIs. HRs were adjusted for age, sex, and CCI. The SES reference categories were low for education and income and living alone for cohabiting status. The proportionality assumption of the Cox regression was assessed using log-minus-log plots and observed Kaplan–Meier curves compared with Cox predicted curves. To investigate underlying mechanisms, stratified analysis was performed for each SES marker on sex, age, and CCI.

All analyses were performed using Stata 18.0 (StataCorp LLC, College Station, TX, USA).

### Ethics, data sharing plan, use of AI, funding, and disclosures

The study was reported to the Danish Data Protection Agency through registration at Aarhus University (record number: AU-2016-051-000001, sequential number 880). For register-based studies conducted in Denmark, approval from an ethics committee is not required. AI tools were not used. Data originate from Danish registries containing personal data, which cannot be shared. This data was handled on secure servers provided by Statistics Denmark, with only aggregated results being presented. The study was funded by Aarhus University, Department of Clinical Epidemiology. The authors declare that they have no conflicts of interest. Complete disclosure of interest forms according to ICMJE are available on the article page, doi: 10.2340/17453674.2025.43189

## Results

### Study population and demographics

**Figure 1:**
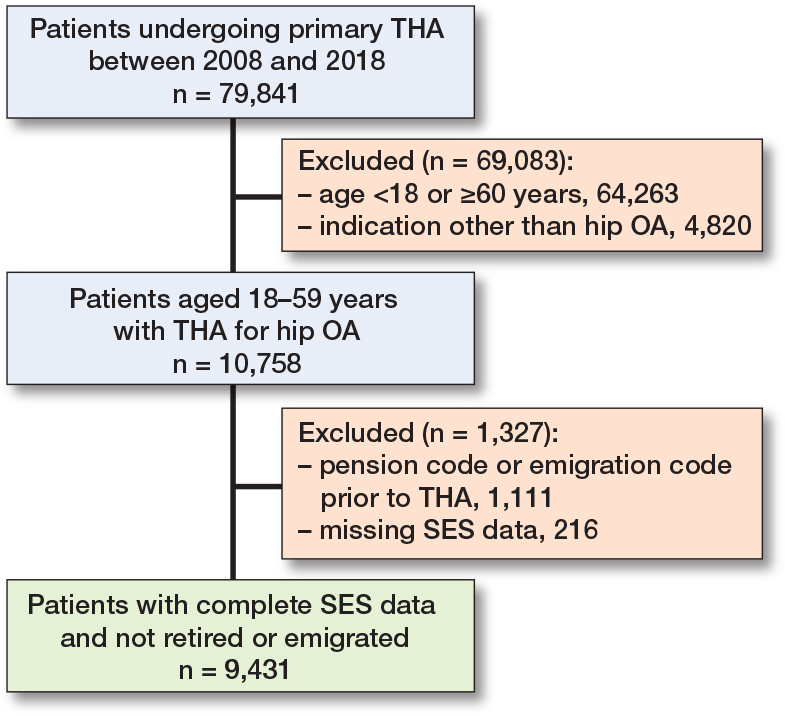
Flowchart describing the inclusion and exclusion process of the study population. THA = total hip arthroplasty. OA = osteoarthritis. SES = socioeconomic status.

The study included 79,841 patients undergoing primary THA due to hip OA from 2008–2018. Patients with indications other than hip OA (6%), aged < 18 or ≥ 60 years of age (80%), missing data on SES markers (0.5%), or coded as retired in DREAM 4 weeks prior to THA (1.5%) were excluded, resulting in a final study population of 9,431 patients (12%) (Figure 1). Patients were predominantly male (56%), on average 55 years old, and the majority had no comorbidities (90%) (Table 1). 6 months prior to THA, most patients were self-supporting or engaged in employment (88%), while a minor proportion were on sick leave (5.8%) or social benefits (6.3%). 4 weeks prior to THA, the proportion of patients on sick leave increased to 12.7% (Table 2). Patient characteristics by SES markers are shown in Table 1.

**Table 1:** Characteristics of the total hip arthroplasty patients overall and by markers of socioeconomic status. Values are count (%) unless otherwise specified

Factor	Overall n = 9,431	Low n = 1,830	Education Medium n = 5,254	High n = 2,347	Low n = 3,144	Income Medium n = 3,144	High n = 3,143	Cohabiting status	
								Living alone n = 1,999	Cohabiting n = 7,432
Sex									
Male	5,242 (56)	1,008 (55)	3,262 (62)	972 (41)	1,604 (51)	1,793 (57)	1,845 (59)	963 (48)	4,279 (58)
Female	4,189 (44)	822 (45)	1,992 (38)	1,375 (59)	1,540 (49)	1,351 (43)	1,298 (41)	1,036 (52)	3,153 (42)
Age									
Median age (IQR)	55 (50–58)	55 (51–58)	54 (50–57)	55 (50–58)	55 (50–58)	55 (50–58)	54 (50–57)	55 (50–58)	55 (50–58)
18–44	829 (8.8)	117 (6.4)	489 (9.3)	223 (10)	322 (10)	292 (9.3)	215 (6.8)	183 (9.2)	646 (8.7)
45–49	1,457 (15)	237 (13)	891 (17)	329 (14)	438 (14)	477 (15)	542 (17)	318 (16)	1,139 (15)
50–54	2,781 (30)	546 (30)	1,577 (30)	658 (28)	861 (27)	911 (29)	1,009 (32)	561 (28)	2,220 (30)
55–59	4,364 (46)	930 (51)	2,297 (44)	1,137 (48)	1,523 (49)	1,464 (47)	1,377 (44)	937 (47)	3,427 (46)
Income per household in euros x 10^3^									
median		75.9	88.2	107	51.8	89.5	129	52.6	96,4
IQR		51.3–95.6	63.9–111	77.8–139	39.5–63.9	81.8–96.7	115–156	39.7–75.3	76.2–121
Charlson Comorbidity Index									
Low	8,506 (90)	1,626 (89)	4,741 (90)	2,139 (91)	2,767 (88)	2,844 (91)	2,895 (92)	1,766 (88)	6,740 (91)
Medium	826 (8.8)	177 (9.7)	462 (8.8)	187 (8.0)	331 (11)	270 (8.6)	225 (7.2)	203 (10)	623 (8.4)
High	99 (1.0)	27 (1.5)	51 (1.0)	21 (0.9)	46 (1.5)	30 (1.0)	23 (0.7)	30 (1.5)	69 (0.9)
Body mass index									
Non-obese	1,604 (17)	238 (13)	877 (17)	489 (21)	454 (14)	421 (13)	729 (23)	357 (18)	1,247 (17)
Obese	767 (8.1)	165 (9.0)	466 (8.9)	136 (5.8)	257 (8.2)	253 (8.0)	257 (8.2)	176 (8.8)	591 (8.0)
Missing	7,060 (75)	1,427 (78)	3,911 (74)	1,722 (73)	2,433 (78)	2,470 (79)	2,157 (69)	1,466 (73)	5,594 (75)

n = number. IQR = interquartile range.

**Table 2:** Preoperative status in the DREAM database at 6 months and 4 weeks prior to THA. Values are count (%)

Status	6 months prior to THA	4 weeks prior to THA
Sick leave	551 (5.8)	1,198 (13)
Self-supporting/other employment	8,290 (88)	7,630 (81)
Social assistance/unemployment benefit	590 (6.3)	603 (6.4)
Total	9,431	9,431

DREAM = Danish Register for Evaluation of Marginalization.

THA = total hip arthroplasty.

### Overall RTW

The overall median time to RTW following THA was 54 days (IQR 7–94) within 24 months (Table 3), with most patients RTW within 6 months (86%, CI 85– 87) (Table 4). The cumulative incidence was 32% at 1 month (CI 31–33) and 70% (CI 69–71) at 3 months. By 12 months, 90% (CI 90–91) had RTW, and this increased to 93% (CI 93–94) at 24 months. Competing risk and censoring accounted for 1.9% of the total events at 24 months.

**Table 3:** Median (interquartile range) time to return to work overall and by markers of socioeconomic status

	Days to return to work
Overall	54 (7–94)
Education	
Low	67 (7–105)
Medium	56 (7–95)
High	42 (1–77)
Income	
Low	67 (7–110)
Medium	62 (21–96)
High	40 (1–69)
Cohabiting status	
Living alone	61 (7–101)
Cohabiting	53 (7–91)

**Table 4:** Cumulative incidence in percentage and (95% confidence interval) of return to work at 1, 3, 6, 12, and 24 months following total hip arthroplasty, overall and by markers of socioeconomic status

Item	1 month	3 months	6 months	12 months	24 months
Overall	32 (31–33)	70 (69–71)	86 (85–87)	90 (90–91)	93 (93–94)
Education					
Low	28 (26–30)	59 (56–61)	77 (75–79)	82 (80–84)	87 (86–89)
Medium	30 (29–31)	69 (68–70)	86 (86–87)	91 (90–91)	93 (93–94)
High	39 (37–41)	80 (78–81)	93 (97–94)	95 (94–96)	97 (96–98)
Income					
Low	26 (25–28)	55 (54–57)	74 (73–76)	81 (79–82)	85 (84–86)
Medium	26 (25–28)	69 (67–71)	89 (87–90)	93 (92–93)	96 (95–96)
High	44 (42–45)	84 (83–85)	95 (94–96)	97 (97–98)	98 (98–99)
Cohabiting status					
Living alone	27 (25–29)	61 (58–63)	79 (77–80)	84 (82–85)	87 (86–89)
Cohabiting	33 (32–34)	72 (71–73)	88 (87–89)	92 (91–92.5)	95 (94–95)

### Education

The time to RTW was 67 days (IQR 7–105) for low education and 42 days (IQR 1–77) for high education. The cumulative incidences for RTW were 28% and 39% at 1 month, 59% and 80% at 3 months, and 87% and 97% at 24 months, among patients with low and high education, respectively. The regression analysis showed an aHR of 1.9 (CI 1.8–2.0) for high education compared with low education (Table 5). In stratified analyses, the aHR for RTW for high compared with low education was 2.2 (CI 2.0–2.4) among male patients and 1.6 (CI 1.5–1.8) among female patients. Patients aged 18–44 years had an aHR of 2.6 (CI 2.0–3.4) for RTW, whereas those aged 55–59 years had an aHR of 1.7 (CI 1.5–1.8) for high compared with low education, with no differences between CCI groups (Table 6).

**Table 5:** Cox proportional hazards regression analyses of the association between markers of socioeconomic status and return to work following total hip arthroplasty within 24 months

Item	Crude hazard ratio (CI)	Adjusted ^a^ hazard ratio (CI)
Education		
Low	Reference	Reference
Medium	1.3 (1.2–1.4)	1.3 (1.2–1.4)
High	1.8 (1.7–1.9)	1.9 (1.8–2.0)
Income		
Low	Reference	Reference
Medium	1.5 (1.4–1.5)	1.5 (1.4–1.5)
High	2.2 (2.1–2.3)	2.2 (2.1–2.3)
Cohabiting status		
Living alone	Reference	Reference
Cohabiting	1.4 (1.3–1.4)	1.3 (1.3–1.4)

CI = 95% confidence interval.

a Adjusted for sex, age, and Charlson Comorbidity Index..

**Table 6:** Cox proportional hazards regression analyses of the association between markers of socioeconomic status and return to work following total hip arthroplasty within 24 months stratified on sex, age, and CCI. All estimates are aHR ^a^ with corresponding 95% confidence intervals

	Sex		Age group				CCI		
	Male	Female	18–44	45–49	50–54	55–59	Low	Medium	High
Education									
Low	Reference								
Medium	1.3 (1.2–1.4)	1.3 (1.2–1.4)	1.8 (1.4–2.3)	1.4 (1.2–1.6)	1.4 (1.2–1.5)	1.2 (1.1–1.3)1.3 (1.2–1.4)	1.3 (1.1–1.6)	1.1 (0.6–2.0)	
High	2.2 (2.0–2.4)	1.6 (1.5–1.8)	2.6 (2.0–3.4)	1.9 (1.6–2.3)	2.1 (1.9–2.4)	1.7 (1.5–1.8)	1.9 (1.8–2.0)	1.8 (1.5–2.3)	1.6 (0.9–3.1)
Income									
Low	Reference								
Medium	1.5 (1.4–1.6)	1.4 (1.3–1.5)	1.5 (1.2–1.7)	1.5 (1.3–1.8)	1.5 (1.4–1.7)	1.4 (1.3–1.5)1.4 (1.4–1.5)	1.4 (1.2–1.7)	2.4 (1.4–4.4)	
High	2.5 (2.4–2.7)	1.9 (1.7–2.0)	2.5 (2.1–3.0)	2.3 (2.0–2.7)	2.4 (2.2–2.7)	2.0 (1.9–2.2)	2.2 (2.1–2.3)	2.2 (1.8–2.6)	2.4 (1.4–4.4)
Cohabiting status									
Living alone	Reference								
Cohabiting	1.5 (1.4–1.6)	1.2 (1.1–1.3)	1.4 (1.2–1.6)	1.5 (1.3–1.7)	1.4 (1.3–1.5)	1.3 (1.2–1.4)	1.3 (1.3–1.4)	1.4 (1.2–1.7)	1.7 (1.0–2.9)

CCI = Charlson Comorbidity Index. aHR = adjusted hazard ratio.

a Estimates for sex, age group, and CCI were adjusted for the 2 other variables.

### Income

**Figure 2:**
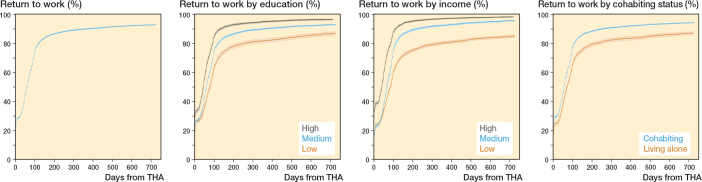
Cumulative incidence with corresponding 95% confidence intervals of return to work (RTW) following primary total hip arthroplasty (THA) within 24 months. Cumulative incidences are visualized for the overall THA population and for categories on education, income, and cohabiting status.

Time to RTW was 67 days (IQR 7–110) for low income and 40 days (IQR 1–69) for high income. The cumulative incidence differed between low and high income at all points of follow-up, with the largest difference occurring at 3 months (Figure 2). Cox regression analysis showed an aHR of 2.2 (CI 2.1–2.3) for high compared with low income. In stratified analyses on sex, differences were found for RTW between males (aHR 2.5, CI 2.4–2.7) and females (aHR 1.9, CI 1.7–2.0) for patients with high income compared with low income, while no significant differences were found while stratifying on age and CCI.

### Cohabiting status

Time to RTW for patients living alone was 61 days (IQR 7–101), and 53 days (IQR 7–91) for patients cohabiting. Cohabiting status affected RTW at all follow-up points, with the largest difference of 11% at 3 months. Cox regression analysis showed an aHR of 1.3 (CI 1.3–1.4) when comparing patients cohabiting with patients living alone. When stratified on sex, the aHR was 1.5 (CI 1.4–1.6) for male and 1.2 (CI 1.1–1.3) for female patients, with no major differences between age and CCI.

### Discussion

We aimed to investigate the association between markers of SES and RTW following primary THA, and whether sex, age, and comorbidity might influence this association. The median time to RTW following THA was 54 days, with most patients returning within 6 months. Markers of low SES, including low education, low income, and living alone, were associated with delayed RTW following THA within 24 months.

Our findings may be explained by several factors affecting a delay in RTW for patients with low SES. Patients with low education and low income are likely to have more physically demanding jobs, thus requiring greater physical recovery than that of a patients with more sedentary jobs before RTW. Patients with a low level of education may face barriers such as lower health literacy, high-risk health behavior, and higher burden of comorbidities [22]. These barriers may work through reduced compliance with postoperative treatment and abilities to understand, communicate, navigate, and advocate within health-related settings, thereby negatively affecting recovery time and RTW. Patients with low income may face poorer living conditions and access to healthcare [22]. Lastly, living alone may negatively affect social support, resulting in a lesser degree of practical and emotional support [23]. Social support is an important factor in coping with pain and rehabilitation after THA, with psychological stress and postoperative complications impeding recovery and delaying time to RTW.

These clinically relevant results may help health professionals in both hospitals and primary care to focus on THA patients in socially vulnerable positions and design interventions to address the barriers they face. While we cannot change the SES of our patients, we can offer a stratified treatment approach. Enhanced rehabilitation programs tailored to the physical and psychological needs of patients with lower SES can be provided. Improving healthcare access through financial and personal support for transportation to rehabilitation facilities can help ensure adherence to follow-up appointments. Social support can be bolstered by establishing local support groups at community centers for practical advice and help with navigating the healthcare system. Employers can facilitate a more gradual RTW plan. Implementing these interventions requires a coordinated effort from healthcare professionals, community centers, and employers to ensure comprehensive support for lower SES patients to successfully RTW following THA. Future studies should focus on designing and testing these interventions.

### Comparison with other studies

Several studies have previously investigated RTW following THA, but few have focused on SES. A Finnish study of 408 public sector employees found that 94% of patients RTW within 1 year, with a mean time of 103 days [17]. However, the generalizability of these findings is limited to public sector employees. A Dutch study by Tilbury et al. reported a mean RTW time of 12.5 weeks and a 90% RTW rate within 1 year among 122 preoperatively working THA patients but found no significant differences based on educational level and living status [18]. Similar results were found by Bohm, who reported 86% RTW after 1 year with no differences in RTW when education and income were assessed [24]. The lack of association between low and high SES could be due to respondents being more likely to have high SES, as loss to follow-up often occurs among patients in the lower SES groups. However, another Dutch study found that 79% of THA patients had RTW after 1 year, with significant differences based on education, showing lower RTW rates for those with low education [25].

The findings of the above-mentioned studies generally align with our findings, showing a 90% RTW rate at 1 year. However, differences in mean time to RTW were observed, with our study finding a median time of 54 days, which is earlier than reported in most studies, likely due to our broader definition of RTW. The inconsistent results regarding SES in previous studies might be caused by small sample sizes and varying data quality on SES markers.

Reproducibility of the study results will be greater for countries with similar social welfare models, such as the Scandinavian countries, or other similar systems used in countries such as France, Germany, and the Netherlands. Countries with a greater lack of a social security system will likely experience a larger inequality in health, affecting patients receiving a THA as well as RTW rates.

### Strengths

This study overcame the limitations of previous studies investigating RTW following THA by including a nationwide cohort of 9,431 patients with complete follow-up over 11 years. This large THA cohort likely represents the population more accurately in terms of SES compared with previous studies, as these mostly have been based on questionnaires, often being more representative of patients in more advantageous socioeconomic positions. Furthermore, by expanding the eligibility criteria on employment status to also include patients on social, unemployment, and sickness benefits, the risk of selection bias was reduced, as patients in these categories would likely be overrepresented in the low SES categories. While some minor misclassification may have occurred due to the large number of codes in DREAM, prior research has shown high positive predictive values (PPV) for the categories self-supporting (98%) and vocational rehabilitation (85%) [15].

Furthermore, our study was based on registry data with high degrees of validity and completeness, contributing with comprehensive and valid information at the individual level. We have analyzed data accounting for competing risks and adjusting for potential confounders, thereby increasing the internal validity of our findings.

### Limitations

Our study also had some limitations. Type of occupation, a potential risk factor for delayed RTW [17], was not included, which could introduce minor confounding due to the ambiguity of previous research. BMI data was missing for 75% of the study population, limiting our ability to assess its confounding effect. The outcome was first-time RTW for 4 consecutive weeks, thus, any potential relapse was not considered. The DREAM does not capture short-term sick leave, possibly leading to minor misclassification of RTW within the first 14 days.

### Conclusion

We found that patients RTW following THA at a median time of 54 days, with the majority returning within 6 months. Low education, low income, and living alone were associated with delayed RTW and no RTW within 24 months. The association between low SES and delayed RTW was more pronounced among male than female patients.

*In perspective,* this association highlights the importance of enhanced focus on THA patients in socially vulnerable positions, to reduce the health and financial implications of delayed RTW.

### Supplementary data

Table S1 and Figure S1 are available as supplementary data on the article page, doi: 10.2340/17453674.2025.43189

## Supplementary Material



## Notes

PA drafted the manuscript. PA, JBP, MGS, SO, EHP, IM, and ABP conceived and designed the study, interpreted the results, and revised the manuscript.

Handling co-editors: Bart A. Swierstra and Philippe Wagner

*Acta* thanks Mikhail Saltychev and Martijn Schotanus for help with peer review of this manuscript.
