# Supplementary material for: Association of socioeconomic status on return to work following primary total hip arthroplasty: a Danish population-based cohort study on 9,431 patients from 2008–2018
Source: Acta Orthop. 2025 Mar 10;96:243–9. doi: 10.2340/17453674.2025.43189 (PMC11894731; doi:10.2340/17453674.2025.43189)
Supplement: Supplementary file 1 [file ActaO-96-43189-s1.pdf]

Supplementary data

Table S1. Codes for public transfer payments included in the DREAM database, and derived groups, used for definition regarding inclusion and outcome

All present and historic codes covering 2008–2018	Description	Outcome category	Inclusion in the study population 4 weeks prior to total hip arthroplasty
No entry, 122, 123, 899	Self-supporting and partially returned	RTW	Include
413, 521, 651, 652, 661, 662, 794	State Education Fund grants and other educational funding	RTW	Include
881	Maternity leave pay	RTW	Include
412, 795	Leave-of absence schemes	Not RTW	Include
750-758, 760-768, 769, 791, 792	Vocational rehabilitation benefit	RTW	Include
115, 771, 772, 773, 774, 779, 781, 782, 796	”Flexible job” and “Light job”	RTW	Include
111-114, 121, 124-126, 211, 212, 213-219, 211, 222, 224, 225, 231, 232, 297, 298, 299, 511, 522, 541, 740, 743-748, 759	Unemployment benefit	Not RTW	Include
130-139, 140-149, 151-153, 160, 163-169, 700, 703-719, 720-729, 730-739, 741, 742	Social assistance, social security payment	Not RTW	Include
611, 621, 622	Voluntary early retirement scheme	Not RTW (retirement—competing risk)	Exclude
783, 793, 797	Anticipatory pension scheme		
998	Public retirement pension		
784, 785, 890-899, 810-818, 870-878	Sickness benefit	Not RTW	Include
997	Emigrated	Not RTW (censoring)	Not applicable
999	Dead	Not RTW (dead—competing risk)	Not applicable

DREAM = Danish Register for Evaluation of Marginalization. RTW = return to work.

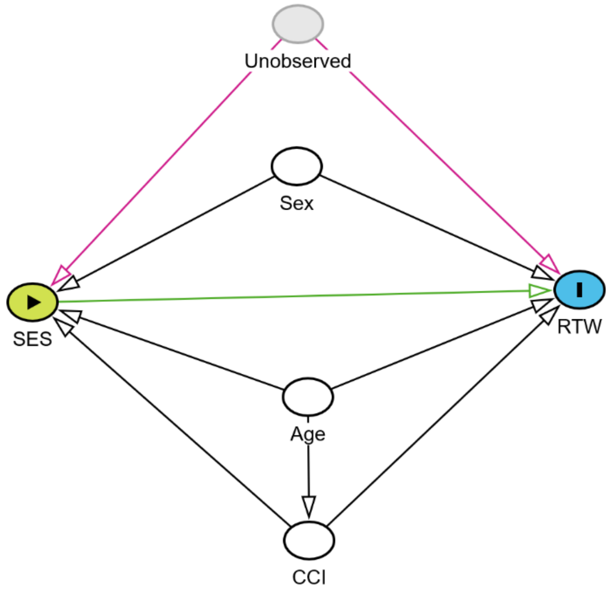

Figure S1. Simplified directed acyclic graph of the analytical design. SES represents the 3 separately analyzed exposures of socioeconomic status: income, education, cohabitating status. Generated using dagitty.net. SES = socioeconomic status. RTW = return to work. CCI = Charlson Comorbidity Index.
